# Efficacy and Safety of Teneligliptin 40 mg in Type 2 Diabetes: A Pooled Analysis of Two Phase III Clinical Studies

**DOI:** 10.1007/s13300-018-0372-x

**Published:** 2018-02-12

**Authors:** Takashi Kadowaki, Kazuyo Sasaki, Manabu Ishii, Miyuki Matsukawa, Yoshiteru Ushirogawa

**Affiliations:** 1 Department of Diabetes and Metabolic Diseases, Graduate School of Medicine, The University of Tokyo, Tokyo, Japan; 2 Ikuyaku. Integrated Value Development Division, Mitsubishi Tanabe Pharma Corporation, Osaka, Japan

**Keywords:** Dipeptidyl peptidase-4 inhibitor, Dose increase, Glycemic control, HbA1c, Post hoc analysis, Teneligliptin, Type 2 diabetes

## Abstract

**Introduction:**

Teneligliptin, an antihyperglycemic agent belonging to the dipeptidyl peptidase-4 inhibitor class, is usually prescribed at a dose of 20 mg/day. In Japan, the dose can be increased to 40 mg/day if needed. We examined the treatment response when the teneligliptin dose was increased from 20 to 40 mg in a post hoc pooled analysis of data from two 52-week, open-label, phase III clinical trials of teneligliptin 20–40 mg/day as monotherapy or combination treatment in Japanese patients with type 2 diabetes.

**Methods:**

In both studies, patients received teneligliptin 20 mg for at least 28 weeks; thereafter the dose was increased if glycemic control was inadequate. The data set for this post hoc analysis comprised those patients whose teneligliptin dose was increased to 40 mg at week 28 (*N* = 204). We assessed (i) the proportion of patients achieving HbA1c reduction after teneligliptin dose increase [≤ − 0.1% change in HbA1c during weeks 28–52 (24 weeks); responders] and (ii) the response to teneligliptin 40 mg according to whether or not patients experienced HbA1c re-elevation (≥ 0.1% increase) during 28 weeks of teneligliptin 20 mg.

**Results:**

Of 204 patients, 108 (52.9%) showed a response to teneligliptin 40 mg (HbA1c change ≤ − 0.1% during weeks 28–52) and had mean (± SD) HbA1c reduction of 0.50 ± 0.44%. Of patients showing re-elevation of HbA1c during treatment with teneligliptin 20 mg, 89/143 (62.2%) achieved HbA1c reduction after dose increase to 40 mg. Logistic regression analyses suggested that change in body weight is one of the parameters linked to HbA1c reduction after dose increase to teneligliptin 40 mg. The incidence of adverse events was not changed after teneligliptin dose increase.

**Conclusion:**

Increasing the dosage of teneligliptin from 20 to 40 mg/day has potential as a well-tolerated and effective option for treating type 2 diabetes.

**Funding:**

Mitsubishi Tanabe Pharma Corporation.

**Electronic supplementary material:**

The online version of this article (10.1007/s13300-018-0372-x) contains supplementary material, which is available to authorized users.

## Introduction

The rising prevalence of diabetes mellitus, especially type 2 diabetes, is a global problem. Japan is one of the nations most affected by this global epidemic; in 2016, a report from the National Health and Nutrition survey in Japan estimated that 10 million adults are strongly suspected of having diabetes and another 10 million who will possibly develop diabetes [1]. Such is the concern about this growing trend, that Japan’s Ministry of Health, Labour and Welfare regarded diabetes as one of most important issues to overcome by 2022, which include reductions in the number of people with elevated blood glucose and diabetic complications and improvement of adherence to treatment [2].

In order to reduce the risk of diabetic complications, guidelines for the management of diabetes set goals for glycemic control [3–5]. In Japan, the current target to avoid diabetic complications is a glycated hemoglobin (HbA1c) less than 7.0%, to be achieved through a combination of diet and exercise therapy, and pharmacotherapy [3]. The choice of orally administered antidiabetic medication is based on the patient’s medical condition and the pharmacological properties of the drug, including its side-effect profile. There is growing evidence that the pathophysiology of diabetes varies between ethnic groups, with β-cell dysfunction leading to reduced insulin secretion being the primary defect in East Asians rather than insulin resistance due to increased adiposity, which characterizes Caucasians [6].

Dipeptidyl peptidase-4 (DPP-4) inhibitors are rapidly becoming a first-line treatment option for patients with type 2 diabetes in Japan [7]. DPP-4 inhibitors act by increasing active glucagon-like peptide-1 (GLP-1) in blood through inhibition of DPP-4, which stimulates glucose-dependent insulin secretion and inhibits glucagon secretion, leading to reduced glucose levels with a low incidence of hypoglycemia [8]. Differences in the efficacy of DPP-4 inhibitors have been observed between Asian and non-Asian patients with diabetes, presumably reflecting differences in the diabetic phenotype [9]. However, there have been reports that prolonged treatment with DPP-4 inhibitors causes re-elevation of HbA1c after initial reductions in some patients [10–13].

Teneligliptin is a novel, orally administered DPP-4 inhibitor with a unique structure having five consecutive rings [14], which provides potent and 24-h glycemic control [15]. Teneligliptin has been prescribed in Japan for the treatment of type 2 diabetes since 2012 at a standard dosage of 20 mg once daily and at a high dosage of 40 mg once daily if the standard dosage is insufficient for glycemic control [16]. The safety and efficacy of teneligliptin 20 mg in Japanese patients with type 2 diabetes has been demonstrated both as monotherapy [15] and in combination with pioglitazone, glimepiride, insulin, and canagliflozin [17–20]. Although our previous study observed similar efficacy with 10, 20, and 40 mg doses [21], a dose-dependent reduction in HbA1c was observed over 24 weeks with teneligliptin 5–40 mg added to metformin therapy [22]. More recently, teneligliptin 40 mg was shown to reduce the mean amplitude of glycemic excursions and to increase minimum glucose concentrations compared with teneligliptin 20 mg in hospitalized patients with type 2 diabetes, suggesting that the higher dose may provide better “quality” glucose control by reducing the risk of a hypoglycemia event [23].

We recently demonstrated the long-term safety and efficacy of teneligliptin as monotherapy or combination treatment in a post hoc pooled analysis of data from two 52-week, open-label clinical studies [24]. These studies allowed titration of the teneligliptin dose from 20 to 40 mg if glycemic control was insufficient at the lower dose. Here we report the results of a second post hoc analysis of data from the same two phase III studies, which we conducted to examine more closely the treatment response occurring when teneligliptin dose was increased from 20 to 40 mg (regardless of monotherapy or combination treatment) at week 28, i.e., the response to teneligliptin 40 mg for 24 weeks. In addition, we evaluated the HbA1c response to teneligliptin 40 mg in patients with or without HbA1c re-elevation during 28 weeks of teneligliptin 20 mg.

## Methods

### Patients and Methodology

This post hoc analysis used data pooled from two long-term, open-label, phase III clinical trials of teneligliptin involving 702 Japanese patients with type 2 diabetes. The design and methodology used in these studies have been described previously [24]. Briefly, in Study 3000-A8 (ClinicalTrials.gov, NCT02314637), patients (*N* = 240) received teneligliptin as monotherapy or in combination with a sulfonylurea, glimepiride, while in Study 3000-A14 (ClinicalTrials.gov, NCT01301833), patients (*N* = 462) received teneligliptin as monotherapy or in combination with a biguanide, glinide, or α-glucosidase inhibitor. The duration of both studies was 52 weeks and all patients received a starting dosage of teneligliptin 20 mg once daily at week 0. In both studies, the dosage of teneligliptin was titrated to 40 mg once daily during weeks 28–40 for those patients who met the criteria for dose increase (HbA1c ≥ 7.3% in Study 3000-A8; ≥ 7.4% in Study 3000-A14) and for whom there were no safety concerns as judged by the investigator. Patients titrated to the higher dose of teneligliptin remained on this dose until week 52. Patients not meeting the criteria for a dose increase remained on teneligliptin 20 mg until week 52.

**Fig. 1:**
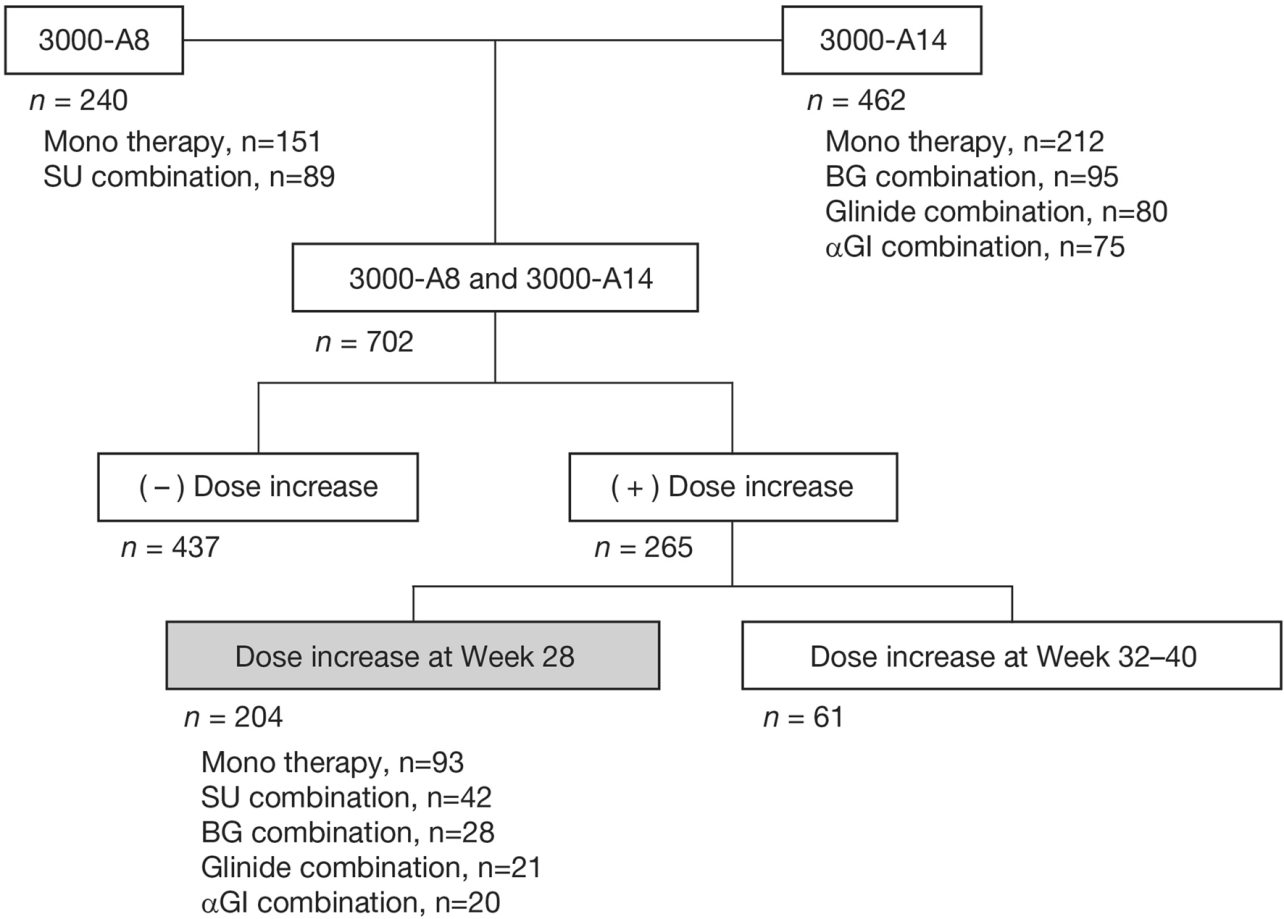
Patient disposition.* α-GI* alpha-glucosidase inhibitor,* BG* biguanide,* SU* sulfonylurea

The data set for this analysis comprised those patients whose teneligliptin dose was increased to 40 mg at week 28 (Fig. 1). Two sub-analyses were conducted. The first sub-analysis assessed the proportion of patients experiencing a reduction in HbA1c with the 40 mg teneligliptin dose (assessed from the change in HbA1c over 24 weeks from week 28 to 52) versus those not experiencing HbA1c reduction during this period. The second sub-analysis assessed the week 52 response to teneligliptin 40 mg after dose increase at week 28 in a subgroup of patients who experienced re-elevation of HbA1c after initial reductions with teneligliptin 20 mg during the first 28 weeks versus those who had no re-elevation of HbA1c during this period. Re-elevation was defined as the difference between the minimum HbA1c level achieved during treatment with teneligliptin 20 mg and HbA1c level at week 28.

These two clinical studies were conducted in compliance with Good Clinical Practice guidelines and the Pharmaceutical Affairs Law in Japan, according to the ethical principles of the Declaration of Helsinki of 1964, as revised in 2008. Informed consent was obtained from all patients before enrollment in those clinical studies. All analyses in the present study were performed on a fully anonymized data set.

### Statistical Analysis

In the first sub-analysis, the week 52 response to teneligliptin 40 mg (change in HbA1c over 24 weeks from week 28 to 52) was stratified by ≤ − 0.1% (response) or > − 0.1% (no response). A subsidiary evaluation using a criterion of ≤ − 0.3% reduction in HbA1c to define a response to teneligliptin 40 mg was also conducted. Logistic regression analysis was carried out to identify parameters associated with HbA1c reduction following teneligliptin 40 mg during weeks 28–52.

In the second sub-analysis, the response to teneligliptin 40 mg during weeks 28–52 was stratified by patients with HbA1c change of ≥ 0.1% during the first 28 weeks of treatment with teneligliptin 20 mg (HbA1c re-elevation) and those with HbA1c change < 0.1% (no re-elevation). A subsidiary evaluation using a change in HbA1c of ≥ 0.3% as the criterion to define HbA1c re-elevation was also conducted. Logistic regression analysis was carried out to identify parameters associated with HbA1c re-elevation during weeks 0–28.

In both sub-analyses, missing data at week 52 were imputed using the last observation carried forward method.

Continuous data were summarized as the number of patients (*n*), mean, and standard deviation (SD), and discrete data were summarized as *n* and percentage values for each category. Statistical tests were two-sided with a 5% significance level, and two-sided 95% confidence intervals (CIs) were calculated. The statistical analysis was performed by Takumi Information Technology Inc. (Tokyo, Japan), using SAS 9.4 (SAS Institute Inc., Cary, NC, USA).

Adverse events (AEs) and adverse drug reactions (ADRs) were classified according to system organ class and preferred term using MedDRA/J version 15.0 (Japanese Maintenance Organization, Tokyo, Japan).

## Results

### Characteristics at Week 0 and Response to Teneligliptin 40 mg from Week 28 to Week 52

In this pooled analysis, there were 204 patients who received a dose increase of teneligliptin at week 28. Of these, 88 patients were from Study 3000-A8 and 116 patients were from Study 3000-A14. The characteristics of these patients at week 0 are shown in Table 1. Just under half the patients (45.6%) received teneligliptin as monotherapy and the remainder received teneligliptin in combination with a sulfonylurea (20.6%), biguanide (13.7%), glinide (10.3%) or α-glucosidase inhibitor (9.8%). Mean (± SD) HbA1c levels in the 204 patients taking teneligliptin 40 mg for 24 weeks during weeks 28–52 were 8.57 ± 0.77% at week 0, 7.93 ± 0.69% at week 28 and 7.85 ± 0.85% at week 52; 10.9% of these patients achieved HbA1c < 7% at week 52 (Table 2 and Figure S1 of supplementary material). Changes in fasting plasma glucose levels and body weight are shown in Table S1 in the supplementary material.

**Table 1:** Patient characteristics at week 0

Characteristic^a^	All patients (*N* = 204)
Male, *n* (%)	131 (64.2)
Age, years	56.3 (9.7)
Body weight, kg	69.31 (14.87)
BMI, kg/m^2^	25.91 (4.49)
Duration of DM, years	7.46 (5.38)
Diabetic complications, *n* (%)	
Any	68 (33.3)
Retinopathy	30 (14.7)
Neuropathy	22 (10.8)
Nephropathy	33 (16.2)
HbA1c, %	8.57 (0.77)
Fasting plasma glucose, mg/dL	171.0 (32.7)
HOMA-R	3.46 (3.59)
HOMA-β	33.66 (84.89)
eGFR, mL/min/1.73 m^2^	87.84 (17.96)
Treatment regimen, *n* (%)	
Teneligliptin monotherapy	93 (45.6)
Combination therapy	
Sulfonylurea	42 (20.6)
Biguanide	28 (13.7)
Glinide	21 (10.3)
α-Glucosidase inhibitor	20 (9.8)

*BMI* body mass index, *DM* diabetes mellitus, *eGFR* estimated glomerular filtration rate, *HbA1c* glycated hemoglobin, *HOMA-β* homeostatic model assessment for beta cell function, *HOMA-R* homeostatic model assessment for insulin resistance, *SD* standard deviation

^a^All values are mean (SD) unless otherwise stated

**Table 2:** HbA1c levels at week 0, week 28 (teneligliptin 20 mg), and week 52 (teneligliptin 40 mg), and proportion of patients achieving HbA1c target < 7% with teneligliptin 40 mg

	All	Response or not to dose increase		Re-elevation or not during 20 mg treatment			
				HbA1c ≥ 0.1% (re-elevation)		HbA1c < 0.1% (no re-elevation)	
		HbA1c ≤ − 0.1% (response)	HbA1c > − 0.1% (no response)	HbA1c ≤ − 0.1% (response)	HbA1c > − 0.1% (no response)	HbA1c ≤ − 0.1% (response)	HbA1c > − 0.1% (no response)
Number of patients	204	108	96	89	54	19	42
HbA1c, %							
Week 0	8.57 (0.77)	8.61 (0.73)	8.53 (0.81)	8.61 (0.75)	8.42 (0.74)	8.66 (0.67)	8.67 (0.87)
Week 28	7.93 (0.69)	8.01 (0.71)	7.83 (0.65)	8.14 (0.71)	7.99 (0.64)	7.42 (0.28)	7.63 (0.61)
Week 52 (LOCF)	7.85 (0.85)	7.51 (0.75)	8.23 (0.80)	7.62 (0.77)	8.34 (0.76)	7.02 (0.39)	8.09 (0.85)
∆ 0–28 weeks	− 0.64 (0.73)	− 0.60 (0.74)	− 0.69 (0.72)	− 0.46 (0.69)	− 0.42 (0.71)	− 1.24 (0.62)	− 1.04 (0.56)
∆ 0–52 weeks (LOCF)	− 0.73 (0.92)	− 1.10 (0.84)	− 0.30 (0.81)	− 0.99 (0.80)	− 0.08 (0.80)	− 1.64 (0.86)	− 0.58 (0.74)
∆ 28–52 weeks (LOCF)	− 0.08 (0.62)	− 0.50 (0.44)	0.39 (0.41)	− 0.53 (0.45)	0.34 (0.32)	− 0.39 (0.41)	0.46 (0.49)
% of patients with HbA1c < 7.0% at week 52	10.9	20.4	–	18.0	–	31.6	–

Data are mean (SD) unless otherwise stated

*HbA1c* glycated hemoglobin, *LOCF* last observation carried forward, *SD* standard deviation

### Subgroup Analysis: Response to Teneligliptin 40 mg

**Fig. 2:**
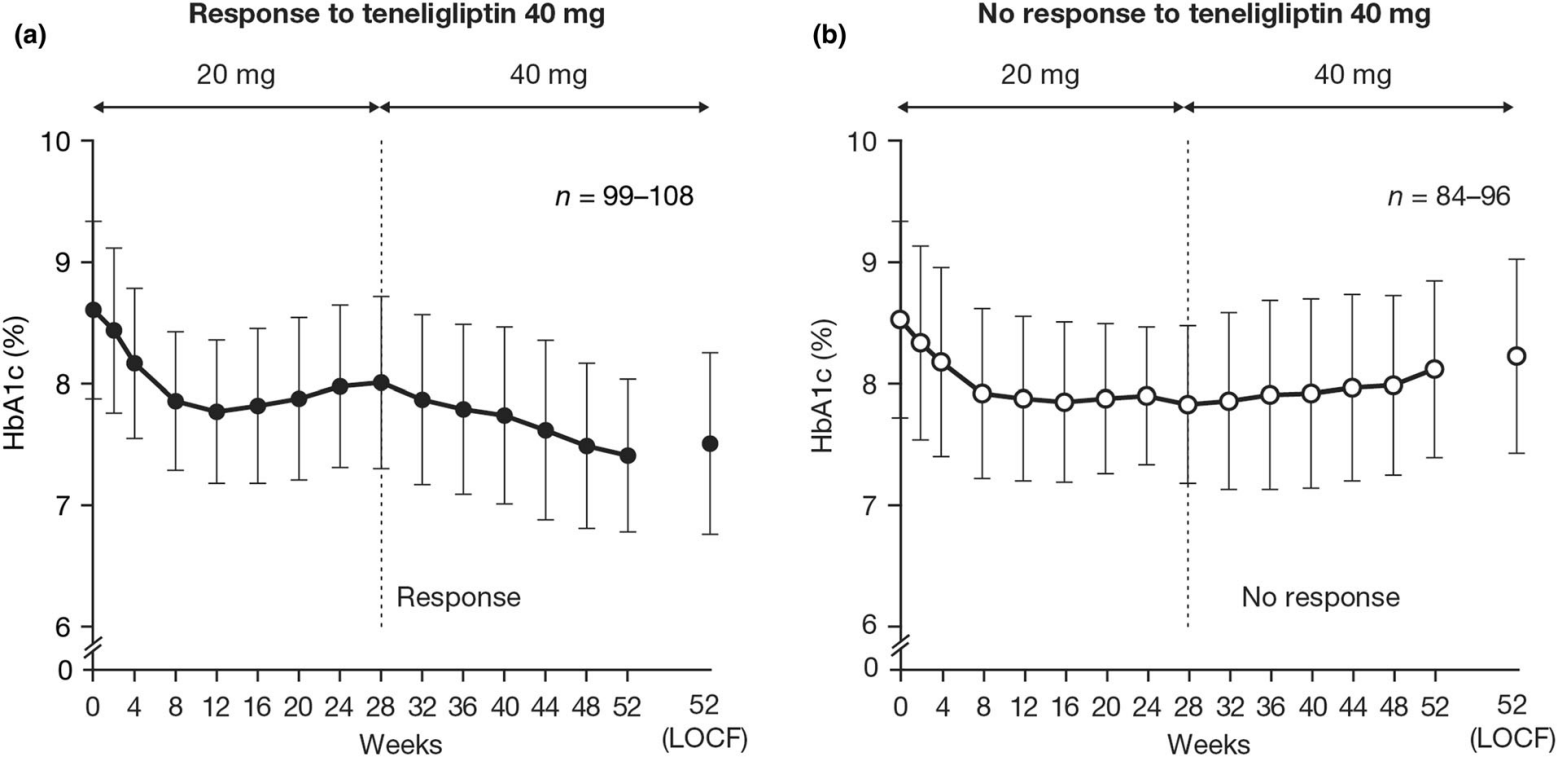
HbA1c levels over time, in subgroups classified by response (**a**) or no response (**b**) at week 52 with teneligliptin 40 mg. Data are mean ± SD.* HbA1c* glycated hemoglobin,* LOCF* last observation carried forward,* SD* standard deviation

Of the 204 patients, 108 (52.9%) showed a response to teneligliptin 40 mg (change in HbA1c of ≤ − 0.1% from week 28 to 52) and 96 patients (47.1%) did not respond (change in HbA1c of > − 0.1% from week 28 to week 52). Figure 2 shows the changing HbA1c profiles of the responders and non-responders to the teneligliptin 40 mg dose. Among the 108 patients who showed a response to teneligliptin 40 mg, 20.4% achieved HbA1c < 7% at week 52 (Table 2). The mean (± SD) change in HbA1c during weeks 28–52 in the patients responding to teneligliptin 40 mg was − 0.50 ± 0.44% (Table 2). Additionally, 74/204 patients (36.3%) achieved a change in HbA1c of ≤ − 0.3% after dose increase; the mean change in HbA1c during week 28–52 was − 0.67 ± 0.44% in these patients.

In order to determine the parameters associated with reduced HbA1c in response to the increased dose of teneligliptin, logistic regression analysis was carried out. Loss of body weight during the increased dosage period (weeks 28–52) was identified (Table 3), and the contribution by this parameter was 4.5% (Table 3). Patients who responded to the increased dose of teneligliptin (HbA1c change of ≤ − 0.1%) had a mean (± SD) body weight loss of 0.93 ± 2.20 kg during weeks 28–52 while non-responders gained 0.13 ± 1.20 kg (*p* < 0.0001; Table S1 in the supplementary material).

**Table 3:** Relationship between HbA1c reduction with teneligliptin 40 mg treatment and various parameters according to logistic regression analysis

Parameter	Odds ratio (95% CI)	*p* value Pr > Chi sq	Partial regression coefficient	*R* ^2^
Result of logistic regression analysis (full model)				
Sex (efficacy rate for female vs male)	1.093 (0.575, 2.076)	0.786	0.000	–
Age	1.024 (0.989, 1.060)	0.175	0.000	–
Duration of DM	0.935 (0.877, 0.996)	0.037	− 0.092	–
HbA1c at week 28	1.721 (0.979, 3.024)	0.059	0.075	–
HOMA-R at week 28	0.897 (0.719, 1.119)	0.335	0.000	–
HOMA-β at week 28	1.005 (0.982, 1.029)	0.681	0.000	–
Body weight, ∆ 28–52 weeks	0.668 (0.541, 0.824)	< 0.001	− 0.208	–
Result of logistic regression analysis (model = stepwise)				
Body weight, ∆ 28–52 weeks	0.672 (0.548, 0.824)	< 0.001	− 0.212	0.0449

*CI* confidence interval,* DM* diabetes mellitus,* HbA1c* glycated hemoglobin,* HOMA-β* homeostatic model assessment for beta cell function,* HOMA-R* homeostatic model assessment for insulin resistance,* Pr > Chi sq* the observed significance probabilities for the Chi square tests,* R*^2^ contribution ratio

### Subgroup Analysis: Response to Teneligliptin 40 mg in Patients with Re-elevation and No Re-elevation of HbA1c During the First 28 Weeks of Treatment

There have been reports that prolonged treatment with DPP-4 inhibitors cause re-elevation of HbA1c [10–13]. Among some patients who showed a reduction in HbA1c after teneligliptin dose increase, there was a tendency to re-elevate HbA1c during administration of the 20 mg dose (Fig. 2). We therefore examined the response to teneligliptin 40 mg among patients with and without re-elevation of HbA1c during the first 28 weeks of teneligliptin 20 mg.

**Fig. 3:**
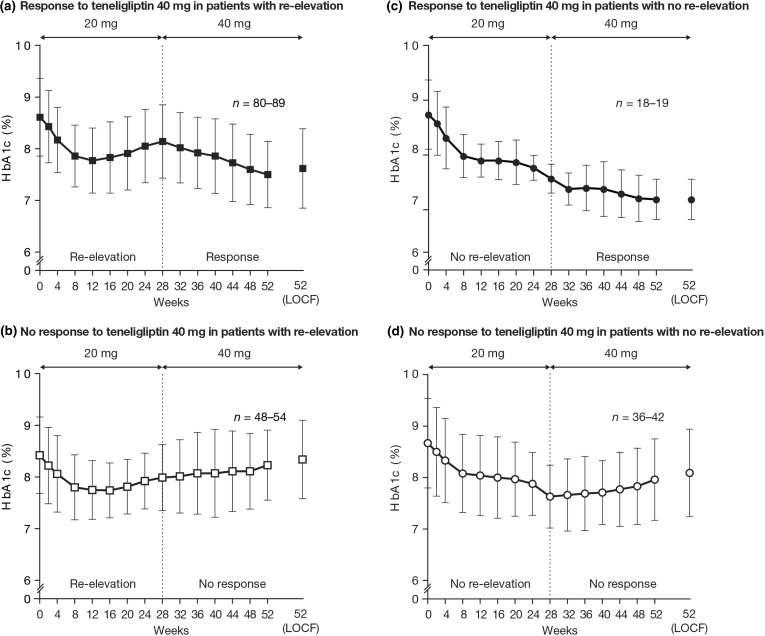
HbA1c levels over time, classified by HbA1c response at week 52 with teneligliptin 40 mg, in subgroups with HbA1c re-elevation or not during weeks 0–28. Data are mean ± SD. Re-elevation during weeks 0–28: ≥ 0.1% was considered re-elevation (**a**, **b**), < 0.1% was not considered re-elevation (**c**, **d**). Response to teneligliptin 40 mg: ≤ − 0.1% was considered response (**a**, **c**), > − 0.1% was no response (**b**, **d**).* HbA1c* glycated hemoglobin,* LOCF* last observation carried forward,* SD* standard deviation

Figure 3 shows the changing HbA1c profiles in patients achieving or not achieving HbA1c reduction with teneligliptin 40 mg at week 52, separated by whether they experienced HbA1c re-elevation (defined as change in HbA1c of ≥ 0.1%) (Fig. 3a, b) or no re-elevation (Fig. 3c, d) during treatment with teneligliptin 20 mg.

Among patients with re-elevation of HbA1c, 89 (62.2%) patients showed a response to teneligliptin 40 mg (Table 2, Fig. 3a). In such patients, mean HbA1c change was – 0.53 ± 0.45% after dose increase and – 0.99 ± 0.80% from week 0 to week 52; 18.0% of these patients achieved HbA1c < 7% at week 52 (Table 2). Among patients without re-elevation of HbA1c, 19 (31.1%) patients showed a response to teneligliptin 40 mg (Table 2, Fig. 3b). In such patients, mean HbA1c change was − 0.39 ± 0.41% after dose increase and − 1.64 ± 0.86% from week 0 to 52; 31.6% of these patients achieved HbA1c < 7% at week 52 (Table 2).

In addition, of 98 patients who experienced re-elevation of HbA1c of ≥ 0.3% during administration of teneligliptin 20 mg, 45 (45.9%) patients achieved a further reduction in HbA1c of ≤ − 0.3% after dose increase; the mean change in HbA1c after dose increase in these patients was − 0.72 ± 0.47%. Among 106 patients with no re-elevation of HbA1c (based on 0.3% criteria), 29 (27.4%) patients achieved a reduction in HbA1c of ≤ − 0.3% after dose increase; the mean change in HbA1c after dose increase in these patients was − 0.59 ± 0.38%.

In order to determine the parameters associated with re-elevated HbA1c during treatment with teneligliptin 20 mg, logistic regression analysis was performed. Increased body weight during weeks 0–28 was identified; the contribution made by this parameter was 4.4% (Table 4).

**Table 4:** Relationship between the re-elevation of HbA1c with teneligliptin 20 mg treatment and various parameters according to logistic regression analysis

Parameter	Odds ratio (95% CI)	*p*-value Pr > Chi sq	Partial regression coefficient	*R* ^2^
Result of logistic regression analysis (full model)				
Sex (re-elevation rate for female vs male)	1.054 (0.535, 2.080)	0.879	0.000	–
Age	1.014 (0.976, 1.053)	0.475	0.000	–
Duration of DM	0.970 (0.909, 1.036)	0.367	0.000	–
HbA1c at week 0	0.895 (0.552, 1.450)	0.652	0.000	–
HOMA-R at week 0	1.097 (0.872, 1.379)	0.430	0.000	–
HOMA-β at week 0	1.002 (0.981, 1.024)	0.836	0.000	–
Body weight, ∆ 0–28 weeks	1.429 (1.164, 1.756)	< 0.001	0.196	–
Result of logistic regression analysis (model = stepwise)				
Body weight, ∆ 0–28 weeks	1.433 (1.178, 1.744)	< 0.001	0.210	0.0441

*CI* confidence interval, *DM* diabetes mellitus, *HbA1c* glycated hemoglobin, *HOMA-β* homeostatic model assessment for beta cell function, *HOMA-R* homeostatic model assessment for insulin resistance, *Pr* *>* *Chi sq* the observed significance probabilities for the Chi square tests, *R*^2^ contribution ratio

### Safety

There was no clear increase observed in the incidence of AEs or adverse drug reactions (ADRs) after teneligliptin dose increase (AEs before and after dose increase: 77.0% and 79.9%; ADRs: 4.9% and 7.4%, respectively) (Table 5). Although the incidence of serious AEs was elevated after increased dosage of teneligliptin, none of these were related to study drug. There were no deaths during the study (Table 5). The AEs that occurred in ≥ 5% of patients before or after teneligliptin dose increase are shown Table S2 of the supplementary material. The most common AE and ADR were nasopharyngitis and hypoglycemia, respectively. There was no clear difference in the incidence of hypoglycemia before or after dose increase (Table 5) or in the incidence of AEs or ADRs in patients taking teneligliptin 40 mg as monotherapy or in combination with other antidiabetic drugs (data not shown).

**Table 5:** Adverse events (all patients, *n* = 204)

Number of patients with AE (%)	Weeks 0–52	Weeks 0–28 (20 mg)	Weeks 28–52 (40 mg)
Any AE	183 (89.7)	157 (77.0)	163 (79.9)
AEs leading to discontinuation	8 (3.9)	1 (0.5)	8 (3.9)
ADR	21 (10.3)	10 (4.9)	15 (7.4)
ADRs leading to discontinuation	1 (0.5)	1 (0.5)	1 (0.5)
Serious AE	12 (5.9)	4 (2.0)	11 (5.4)
Serious ADR	0 (0.0)	0 (0.0)	0 (0.0)
Death	0 (0.0)	0 (0.0)	0 (0.0)
Hypoglycemia	5 (2.5)	3 (1.5)	2 (1.0)

Cases where events continued before and after increased dosage were recorded as one case before and after

*ADR* adverse drug reaction, *AE* adverse event

## Discussion

Our previous post hoc analysis of pooled data from two phase III open-label clinical studies showed that long-term use of teneligliptin 20–40 mg/day as monotherapy or combination therapy was well tolerated and significantly improved glycemic control in Japanese patients with type 2 diabetes [24]. A randomized, double-blind, placebo-controlled study conducted in European patients with type 2 diabetes showed a dose-dependent reduction in HbA1c over 24 weeks with teneligliptin at doses of 5–40 mg/day added to stable metformin therapy [22]. Reductions in HbA1c observed with the teneligliptin 20 and 40 mg doses at week 24 were − 0.48% and − 0.63%, respectively (placebo-adjusted) [22]. This is in contrast to another of our previous studies [21] in which we observed a similar magnitude of response to teneligliptin at doses of 10–40 mg.

Teneligliptin administered at the standard dosage of 20 mg/day has been shown to improve 24-h blood glucose control in Japanese patients with type 2 diabetes [15]. Postprandial glucose excursions, 24-h mean glucose values, and fasting plasma glucose levels were all significantly reduced with teneligliptin compared with placebo. A recent pilot study using continuous glucose monitoring to compare the effects of teneligliptin 20 and 40 mg doses in hospitalized patients with type 2 diabetes observed a significant dose-dependent reduction in the mean amplitude of glycemic excursions and maximum glucose levels [23]. High-dose teneligliptin also significantly increased minimum glucose concentrations compared with the standard dose, suggesting that teneligliptin 40 mg may provide better “quality” glucose control by reducing the risk of hypoglycemia, and may suppress diabetic complications arising from micro- and macroangiopathies [23].

The present post hoc analysis used data from the same two studies that we used previously [24]. This time we aimed to examine more closely the treatment response when the teneligliptin dose was increased from 20 to 40 mg/day at week 28 in patients who had inadequately controlled blood glucose levels with teneligliptin 20 mg. We observed a response to teneligliptin 40 mg in 52.9% of patients according to our criterion of a ≤ − 0.1% reduction in HbA1c over weeks 28–52. Average HbA1c reduction in these patients over this time period was − 0.50% and approximately one in five of these patients (20.4%) achieved HbA1c < 7% at week 52. Logistic regression analysis found that loss of body weight during the increased dosage period (weeks 28–52) may be linked to the HbA1c response to the increased dose of teneligliptin, although this parameter did not make a major contribution (< 5%). On the basis of reports that DPP-4 inhibitors have a neutral effect on body weight [5, 25], our current result supports evidence that self-control of body weight is one of the factors that can help to maintain glycemic control in patients with diabetes [3–5], alongside glycemic control via the inhibition of DPP-4. Adipose tissue is one of the tissues that produces DPP-4 and obese subjects have been shown to have elevated expression of DPP-4 in visceral fat and higher circulating levels of DPP-4 than lean controls [26]. Although there were no clear differences in body weight at week 28 between HbA1c responders and non-responders in the present study, the reduction in HbA1c levels observed as a result of the increased dose of teneligliptin may be attributable to greater inhibition of DPP-4.

Some studies have reported re-elevation of HbA1c levels during long-term use of DPP-4 inhibitors [10–13]. In our study, twice as many patients with HbA1c re-elevation ≥ 0.1% during treatment with teneligliptin 20 mg showed a response to teneligliptin 40 mg compared to those without re-elevation (62.2% vs 31.1%, respectively). The increase in body weight was selected as the related parameter for HbA1c re-elevation in the logistic regression analysis, although the contribution was not major (< 5%). Other studies have reported increased body weight as a parameter contributing to re-elevation of blood glucose levels after administration of DPP-4 inhibitors [10, 12, 13]. In the study by Kanamori and Matsuba, inadequate compliance with diet and exercise therapy was shown to be an independent determinant of re-elevation of HbA1c after DPP-4 inhibitor treatment, along with body weight [13]. Therefore, re-elevation of HbA1c may be linked to increases in body weight during treatment with teneligliptin 20 mg, and one of the parameters contributing to increased body weight is likely to be an unhealthy lifestyle, including poor management of diet and exercise. If body weight increases, there is a possibility that tissues will become resistant to insulin, even if it is secreted in response to GLP-1 stimulation. Additionally, patients with high BMI or insulin resistance have been found to have high concentrations of serum DPP-4 [26–28]. The results of a 12-week clinical trial of teneligliptin revealed that at week 12 the mean percentage inhibition of plasma DPP-4 activity (measured pre-dose) following teneligliptin 20 and 40 mg once daily was 61.1% and 73.3%, respectively, in Japanese patients with type 2 diabetes [21]. However, high concentrations of blood DPP-4 are believed to lessen the glucose-lowering effects of DPP-4 inhibitors [27], and therefore higher dose of DPP-4 inhibitor may be necessary to inhibit the DPP-4 activity.

Although AEs were observed in 79.9% of patients and ADRs in 7.4% of patients following administration of teneligliptin 40 mg for 24 weeks, there was no clear increase in incidence compared to before the dose increase. It has been reported that DPP-4 and sulfonylurea combinations therapy increase the risk of hypoglycemia [24, 29]. However, there was no clear difference in the incidence of hypoglycemia before or after teneligliptin dose increase with/without sulfonylurea in this study (data not shown).

A wide variety of drugs are available to treat type 2 diabetes. It is important that therapeutic agents are selected on the basis of the patient’s condition and the various properties of the drugs. Therapeutic options are often reduced in certain patient populations such as the elderly patients and those with impaired renal function. Teneligliptin can be administered to patients with renal dysfunction, including patients on dialysis, without the need for dose adjustment [30, 31]. Our results may provide valuable information for diabetes therapy in patients, including those with limited therapeutic options. Further studies should examine the characteristics of patients who will acquire the most benefit from a high dose of teneligliptin.

There are several potential limitations to this post hoc pooled analysis. These include the open-label design of the studies and the relatively small sample size. In addition, patients with a high risk of cardiovascular disease and with severe diabetic complications were excluded from the studies [24]. It is possible that patients who had poor glycemic control at an early phase were selected, because the study analyzed patients who received an increased dose of teneligliptin from week 28. The current analysis does not take into account the seasonal fluctuations in HbA1c [32] and any changes in adherence to teneligliptin and/or concomitant agents that may have occurred after dose increase. Finally, this analysis did not evaluate the effects of teneligliptin in combination with another therapeutic agent for type 2 diabetes such as insulin, thiazolidinedione, and sodium glucose co-transporter 2 inhibitors.

## Conclusions

This post hoc analysis provides evidence that increasing the dosage of teneligliptin from 20 to 40 mg/day has potential as a well-tolerated and effective option for treating Japanese patients with type 2 diabetes.

## Electronic supplementary material

Below is the link to the electronic supplementary material.

Supplementary material 1 (PDF 217 kb)

Supplementary material 2 (PDF 103 kb)
