# Supplementary material for: Efficacy and Safety of Teneligliptin 40 mg in Type 2 Diabetes: A Pooled Analysis of Two Phase III Clinical Studies
Source: Diabetes Ther. 2018 Feb 12;9(2):623–36. doi: 10.1007/s13300-018-0372-x (PMC6104275; doi:10.1007/s13300-018-0372-x)
Supplement: Supplementary file 1 — Supplementary material 1 (PDF 217 kb) [file 13300_2018_372_MOESM1_ESM.pdf]

Efficacy and Safety of Teneligliptin 40 mg in Type 2 Diabetes: A Pooled Analysis of Two Phase III Clinical Studies

2 型糖尿病患者へのテネリグリプチン 40 mg 投与の有効性と安全性：2 つの第Ⅲ相開発治験の統合解析(和訳)

抄録

背景と目的：DPP-4 阻害薬である高血糖改善薬テネリグリプチンは，通常 1 日 1 回 20 mg を処方され，日本では必要に応じて 40 mg に増量が可能である．2 つの 52 週間非盲検第Ⅲ相開発治験(日本人 2 型糖尿病患者にテネリグリプチン 20−40 mg を単独または併用)のデータについてポストホック統合解析を行い，テネリグリプチンの用量を 20 mg から 40 mg に増量した際の反応を検討した．

方法：2 つの治験では，患者はテネリグリプチン 20 mg を 28 週間以上投与された．その後血糖コントロールが不十分であった場合に用量を増量した．このポストホック解析では 28 週時点で 40 mg に増量した患者(N=204)を対象とした．我々は以下について検討した．(i)テネリグリプチン増量後に HbA1c 値の低下(28 週時から 52 週時までの 24 週間の HbA1c 値の変化量≤−0.1%)が認められた患者(Responder)の割合，(ii)テネリグリプチン 20 mg を 28 週間投与している間に HbA1c 値の再上昇(0.1%以上の増加)があった症例となかった症例における，40 mg に対する反応性.

結果：204 例のうち 108 例(52.9%)がテネリグリプチン 40 mg に反応し(28 週時から 52 週時の間の HbA1c 値の変化量≤−0.1%)，この間に平均 HbA1c 値が 0.50±0.44%低下した．20 mg 投与期間中に HbA1c 値の再上昇があった症例のうち，89/143 例(62.2%)で 40 mg 増量により HbA1c 値の低下が認められた．ロジスティック解析の結果から，テネリグリプチン 40 mg への増量後における HbA1c 値の低下には，体重の変化が関与している可能性が示唆された．増量後の薬剤に関連した有害事象の発現割合の変化は認められなかった.

結論：テネリグリプチンを 20 mg から 40 mg に増量することは，2 型糖尿病治療において忍容性があり効果的な選択肢になりうる.

背景

糖尿病，特に 2 型糖尿病の増加は世界的に問題となっている．日本もその国の 1 つであり，2016 年の厚生労働省国民健康・栄養調査では糖尿病が強く疑われる者，糖尿病の可能性を否定できない者はそれぞれ 1000 万人と推定されている[1]．こうした懸念から，厚生労働省は，高血糖および糖尿病性合併症を有する患者を減少させることや，糖尿病の治療継続率を向上させることなどを，2022 年までに克服すべき重要な課題の 1 つとして挙げている[2].

糖尿病性合併症のリスク低減を目指し，糖尿病治療ガイドラインでは血糖値のコントロー

ル目標を設定している[3-5]．そのため日本の血糖コントロール目標は，食事・運動療法および薬物療法の組み合わせにより HbA1c 値 7.0%未満に設定されている[3]．経口糖尿病治療薬は患者の病態や副作用も含めた薬剤の作用特性を考慮して選択されている．さまざまな人種における糖尿病病態生理学のエビデンスの蓄積から，東アジア人では，白人のような肥満によるインスリン抵抗性よりも，インスリン分泌低下につながるβ細胞の機能不全を起こしやすいことがわかってきた[6]．

近年，DPP-4 阻害薬は日本での 2 型糖尿病治療において急速に第一選択薬となった[7]．DPP-4 阻害薬は DPP-4 の阻害を介して血中の活性型 GLP-1 を増加させ，グルコース依存性のインスリン分泌を促進するとともにグルカゴン分泌を抑制する．このため，DPP-4 阻害薬は低血糖を起こしにくい血糖低下薬である[8]．アジアの糖尿病患者と非アジアの糖尿病患者の間に DPP-4 阻害薬の効果の違いが認められており，糖尿病のフェノタイプの違いによるものであろうと考えられている[9]．しかしながら，DPP-4 阻害薬を長期間投与すると，低下した HbA1c 値が再び上昇する患者が存在することが報告されている[10-13]．

新規の経口 DPP-4 阻害薬テネリグリプチンは, 5 つの環がつながったユニークな構造をもち[14]，強力でかつ 24 時間持続する血糖低下作用を有する[15]．テネリグリプチンは，日本では 2012 年から 2 型糖尿病治療薬として処方されている．通常，1 日 1 回 20 mg を投与し，効果が不十分な場合には，1 日 1 回 40 mg に増量することが可能である[16]．日本人 2 型糖尿病患者でのテネリグリプチン 20 mg の安全性と有効性は，単独療法[15]およびピオグリタゾン，グリメピリド，インスリン，カナグリフロジンとの併用療法で確認されている[17-20]．これまで我々が行った試験では 10 mg，20 mg および 40 mg は同程度の薬効を示しているものの[21]，メトホルミンとの併用では 24 週間以上テネリグリプチン 5–40 mg を投与することによって，用量依存的な HbA1c 値の低下が認められている[22]．最近の報告では，入院 2 型糖尿病患者にテネリグリプチン 40 mg を投与すると，20 mg 投与時よりも，血糖値の変動幅の減少や最低血糖値の上昇が認められ，高用量のテネリグリプチンが低血糖を防ぎながら血糖コントロールの質をより良くしている可能性が示された[23]．

最近我々は，テネリグリプチンの 2 つの 52 週間非盲検開発治験のポストホック統合解析により，単独または併用療法における本剤の長期の安全性と有効性を示した[24]．これらの治験ではテネリグリプチン 20 mg で効果が不十分な場合に 40 mg に増量することを可能としている．そこで，我々はこれら 2 つの第Ⅲ相開発治験データを用いて 2 つ目のポストホック解析を行い，単独，併用問わず 28 週時点でテネリグリプチンを 20 mg から 40 mg に増量し 24 週間投与したときの効果を検討した．さらに，20 mg を 28 週間投与している間に HbA1c 値の再上昇があった症例となかった症例における，40 mg 増量後の HbA1c 値の変化について検討した．

方法

対象患者と方法

このポストホック解析では，日本 2 型糖尿病患者 702 例を対象としたテネリグリプチンの 2 つの長期・非盲検・第Ⅲ相開発治験の統合データを用いた．これらの治験のデザインと方法は既報のとおりである[24]．概略を以下に示す．3000-A8 試験(ClinicalTrials.gov., NCT02314637)では，患者(N=240)は単独または SU 薬グリメピリドとの併用にてテネリグリプチンが投与され，3000-A14 試験(ClinicalTrials.gov., NCT01301833)では，患者(N=462)は単独またはビグアナイド薬，グリニド薬，または α–グルコシダーゼ阻害薬との併用にてテネリグリプチンが投与された．いずれの治験も観察期間は 52 週間であり，投与 0 週時のテネリグリプチン投与用量は 20 mg/回/日であった．いずれの治験も増量クライテリア(3000-A8 試験：HbA1c ≥7.3%，3000-A14 試験：≥7.4%)を満たし，かつ安全性に問題がないと判断された患者は，28 週から 40 週までのいずれかの時点で 40 mg/回/日に増量された．テネリグリプチンを増量した患者では 52 週までこの用量が維持された．増量のクライテリアを満たさなかった患者は 52 週まで 20 mg の投与が継続された．

今回の解析に用いたデータセットは，これらの患者のうち 28 週時点でテネリグリプチンを 40 mg に増量した患者から構成された(Fig. 1)．2 つのサブ解析を実施した．1 つ目の解析では，28 週時から 52 週時までの 24 週間に，テネリグリプチン 40 mg 投与により HbA1c 値が低下した患者とそうでない患者の患者割合を評価した．2 つ目の解析では，テネリグリプチン 20 mg を最初に 28 週間投与している間に，HbA1c 値の低下とその後 HbA1c 値の再上昇があった患者とそうでない患者において，28 週時点でテネリグリプチン 40 mg を投与してから 52 週時までの HbA1c 値の変化を評価した．再上昇は，20 mg 投与期間中に得られた最も低い HbA1c 値と 28 週時点の HbA1c 値の差で定義した．

両試験は，2008 年改訂版ヘルシンキ宣言の精神を尊重し，医薬品の臨床試験の実施の基準に関する省令(GCP)並びに関連法規制を遵守して実施された．これらの治験に登録する前に，全ての患者からインフォームドコンセントを取得した．本検討の解析は，すべて匿名化がされているデータセットを用いた．

解析方法

1 つ目のサブ解析では，テネリグリプチン 40 mg に対する 52 週時点での反応(28 週時から 52 週時までの 24 週間の HbA1c 変化量)が≤–0.1%(response)の場合と>–0.1%(no response)の場合とに層別した．また，副次的に，反応性の基準を HbA1c ≤–0.3%として評価した．28 週時から 52 週時までの 40 mg 投与による HbA1c 値の低下と各パラメーターとの関係性を検討するため，ロジスティック回帰分析を実施した．

2 つ目のサブ解析では，テネリグリプチン 20 mg を最初に 28 週間投与している間に，0.1%以上の HbA1c 値の再上昇があった患者とそうでなかった患者に層別し，28 週時から 52 週時までの 40 mg 投与に対する反応性について検討した．また，副次的に，再上昇の基準を HbA1c ≥0.3%として評価した．0 週時から 28 週時までの HbA1c 値の再上昇と各パラメーターとの関係性を検討するため，ロジスティック回帰分析を実施した．

これらのサブ解析では，52 週時のデータが欠損値の場合は，Last Observation Carried

Forward 法により補完した．

連続データは患者数(n)，平均値，標準偏差(SD)にて示し，非連続データは各カテゴリーでの例数および割合で示した．有意水準を 5%とした両側検定を行い，両側 95%信頼区間を算出した．統計解析は SAS 9.4 を用いて株式会社タクミインフォメーションテクノロジーが実施した．

有害事象および副作用用は，MedDRA/J version 15.0 を用いて，器官別大分類および基本語に分類した．

結果

患者の患者背景と 28 週時から 52 週時までテネリグリプチン 40 mg を投与した際の反応

このプール解析において，28 週時にテネリグリプチンを増量した患者は 204 例であった．このうち，3000-A8 試験では 88 例，3000-A14 試験では 116 例であった．これらの患者の患者背景を Table 1 に示す．約半数(45.6%)がテネリグリプチン単独投与，残りが SU 薬(20.6%), ビグアナイド薬(13.7%), グリニド薬(10.3%)または α–グルコシダーゼ阻害薬(9.8%)との併用投与であった．28 週時から 52 週時まで 40 mg を投与した 204 例の HbA1c 値(平均±標準偏差)は，0 週時 8.57±0.77%，28 週時 7.93±0.69%，52 週時 7.85±0.85%であり，52 週時では 10.9%の患者が HbA1c <7%を達成した(Table 2，Figure S1)．空腹時血糖値と体重の変化は Table S1 に示す．

サブグループ解析：テネリグリプチン 40 mg への反応

204 例のうち，テネリグリプチン 40 mg に対する反応(28 週時から 52 週時までの HbA1c 変化量 ≤–0.1%)は 108 例(52.9%)で認められ，96 例(47.1%)では HbA1c 値の低下は認められなかった(28 週時から 52 週時までの HbA1c 変化量>–0.1%)．28 週時にテネリグリプチンを増量して 52 週時に反応があった患者とそうでなかった患者の HbA1c 値の変化を Figure 2 に示す．テネリグリプチン 40 mg に対する反応があった 108 例では，20.4%の患者が 52 週時に HbA1c <7%を達成した(Table 2)．これらの患者の 28 週時から 52 週時までの HbA1c 値の変化量の平均値は–0.50±0.44%であった(Table 2). 加えて，74/204 例(36.3%)では，増量後 HbA1c 変化量が ≤–0.3%であり，28 週時から 52 週時までの HbA1c 値の変化量の平均値は–0.67±0.44%であった．

増量したテネリグリプチンに対する反応との関連因子を検討するためロジスティック回帰分析を実施した. 増量期間中(28 週時から 52 週時まで)の体重低下が因子として選択され，その寄与率は 4.5%であった(Table 3)．テネリグリプチン増量後に HbA1c 値が低下（増量後の HbA1c 変化量 ≤–0.1%）した患者では，28 週時から 52 週時までに体重が 0.93±2.20 kg 減少し，HbA1c 値が低下しなかった患者では 0.13±1.20 kg 増加した(p<0.0001, Table S1).

サブグループ解析：最初の 28 週間に HbA1c 値の再上昇を認めた患者および再上昇を認めなかった患者における，テネリグリプチン 40 mg に対する反応

長期間の DPP-4 阻害薬の投与によって HbA1c 値の再上昇が起こることが報告されている

[10-13]．テネリグリプチンを増量した後に HbA1c 値が低下した一部の患者でも，20 mg 投与期間中に HbA1c 値の再上昇する傾向があった(Fig. 2)．そこで，20 mg を投与した 28 週間に HbA1c 値の再上昇があった患者となかった患者について，40 mg 投与後の効果について検討した．

20 mg 投与期間中に 0.1%以上の HbA1c 値の再上昇を認めた患者(Fig. 3a および 3b)とそうでない患者(Fig. 3c および 3d)について, 28 週時に増量した後 52 週時に HbA1c 値の低下を認めた場合と認めなかった場合の HbA1c 値の変化を Figure 3 に示す．

HbA1c 値の再上昇があった患者のうち，89 例(62.2%)がテネリグリプチン 40 mg 投与後の HbA1c 値の低下が認められた(Table 2, Fig. 3a)．このような患者では増量後の HbA1c 値の変化量の平均値は–0.53±0.45%で，0 週時から 52 週時までの変化量の平均値は–0.99±0.80%，18.0%の患者が 52 週時点で HbA1c <7%を達成した(Table 2)．HbA1c 値の再上昇が認められなかった患者のうち，19 例(31.1%)でテネリグリプチン 40 mg 投与後の HbA1c 値の低下が認められた(Table 2, Fig. 3b)．このような患者では，増量後の HbA1c 値の変化量の平均値は–0.39±0.41%, 0 週時から 52 週時までの変化量の平均値は–1.64±0.86%であり，31.6%の患者が 52 週時点で HbA1c <7%を達成した(Table 2).

20 mg 投与期間中に 0.3%以上の HbA1c 値の再上昇を認めた 98 症例のうち, 45 例(45.9%)では増量後に HbA1c 値が 0.3%以上低下し, 増量後の変化量の平均値は–0.72±0.47%であった．0.3%以上の HbA1c 値の再上昇がなかった患者(106 例)では，29 例(27.4%)が増量後に HbA1c 値が 0.3%以上の低下を認め，増量後の変化量の平均値は–0.59±0.38%であった．

テネリグリプチン 20 mg 投与期間中の HbA1c 値の再上昇との関連因子を検討するため，ロジスティック回帰分析を行ったところ，0 週時から 28 週時までの体重の増加が因子として選択され，その寄与率は 4.4%であった(Table 4).

安全性

テネリグリプチンを 40 mg に増量した後に, 有害事象や副作用の発現の明らかな増加は認められなかった(増量前後の有害事象：77.0%および 79.9%; 副作用：4.9%および 7.4%)(Table 5)．増量後に重篤な有害事象の発現が増えたが，テネリグリプチンに起因する事象は認められなかった．治験期間中の死亡例はなかった(Table 5)．増量前後のいずれかの期間で 5%以上の患者で発現した有害事象を Table S2 に示す．もっとも多く認められた有害事象および副作用は各々鼻咽頭炎および低血糖であった．増量前後で低血糖の有害事象発現割合の明らかな違いは認められなかった(Table 5)．また，単独療法と併用療法とで，テネリグリプチン 40mg による有害事象や副作用の発現割合に明らかな違いは認められなかった．

考察

我々はこれまでに，テネリグリプチンの 2 つの第Ⅲ相非盲検開発治験のポストホック統合

解析により，日本人 2 型糖尿病患者に対する単独または併用療法におけるテネリグリプチン(20–40 mg/日)の長期の安全性と有効性を示した[24]．欧州の 2 型糖尿患者を対象にした二重盲検プラセボ対照試験において，メトホルミンにテネリグリプチン(5–40 mg/日)を 24 週間追加投与することで，用量依存的に HbA1c 値が低下することが示された[22]．投与 24 週時において, テネリグリプチン 20 mg および 40 mg により, HbA1c 値がそれぞれ 0.48%, 0.63%低下した(対プラセボ差)[22]．一方，これまでの他の検討では，テネリグリプチンの 10–40 mg は同等の効果を示した[21]．

日本人 2 型糖尿病患者に対して標準投与量である 20 mg のテネリグリプチンを投与すると，血糖が 24 時間コントロールされた[15]．プラセボに比較して，テネリグリプチンは食後高血糖，24 時間平均血糖値および空腹時血糖値をいずれも有意に低下させた．持続血糖モニタリングシステムを用いてテネリグリプチン 20 mg と 40 mg の比較を行った最近のパイロット研究では，入院 2 型糖尿病患者の平均血糖変動幅と最高血糖値が用量依存的に低下することが示された[23]．また，高用量では最低血糖値が上昇したことから，テネリグリプチン 40 mg が低血糖発現のリスクや細小／大血管障害に起因する糖尿病合併症のリスクを軽減し，より質のよい血糖コントロールが得られる可能性があることが示唆された[23]．

今回のポストホック解析では以前用いた 2 つの治験データ[24]を使用した. 今回, 我々は, テネリグリプチン 20 mg で十分な血糖コントロールが得られず, 28 週時点で 40 mg に増量した患者を対象に，治療効果をより詳細に検討した．28 週から 52 週までにテネリグリプチン 40 mg 投与により HbA1c 値が 0.1%以上低下した患者の割合は 52.9%であった．これらの患者の HbA1c 値の変化量の平均値は–0.50%であり，52 週時点で約 1/5(20.4%)の患者が HbA1c<7.0%を達成した．ロジスティック解析の結果，寄与は大きくない(<5%)ものの，増量期間中の体重低下がテネリグリプチン増量後の HbA1c 値の低下と関連がある可能性が示唆された．DPP-4 阻害薬自体は, 体重に対する影響は小さい[5,25]という報告に基づけば, 今回の結果は，糖尿病患者での血糖コントロールを維持するうえで，DPP-4 阻害による血糖コントロールと共に，患者自身による体重のコントロールも血糖コントロールに寄与する要因の 1 つであること[3-5]を支持している．脂肪組織は DPP-4 産生組織の 1 つであり，肥満患者では内臓脂肪での DPP-4 発現の上昇と血中 DPP-4 量の上昇が報告されている[26]. 今回の研究では増量に対して反応した患者とそうでない患者とで 28 週時の体重に明らかな差は認められなかったが, テネリグリプチンを増量したことでさらに DPP-4 活性を阻害し, HbA1c 値の低下が得られた可能性が考えられる.

いくつかの研究では DPP-4 阻害薬の長期使用における HbA1c 値の再上昇が報告されている[10-13]．我々の検討では，20 mg を投与期間中に 0.1%以上の再上昇が認められた患者では，再上昇がなかった患者に比較して，40 mg に反応した患者数が 2 倍多かった(62.2% vs 31.1%)．ロジスティック解析の結果，寄与率は大きくないものの(<5%)，体重増加が HbA1c 値の再上昇と関連がある可能性が示唆された．他の研究では，DPP-4 阻害薬投与後の血糖値の再上昇に寄与する因子として体重増加が報告されている[10,12,13]．さらに，Kanamori

と Matsuba は体重増加とともに，患者の食事・運動療法遵守が不十分であることも DPP-4 阻害薬投与後の HbA1c 値の再上昇の独立因子であると報告した[13]．したがって，テネリグリプチン 20 mg 期間中の HbA1c 値の再上昇は，体重増加と関連している可能性があり，体重増加の一因として食事や運動のマネジメント不良など不健康な生活習慣が考えられる．GLP-1 によりインスリンが分泌したとしても，体重が増加した状態では組織はインスリン抵抗性の状態である可能性がある．加えて，BMI が高い患者やインスリン抵抗性を有する患者では，血中 DPP-4 濃度が高いことが示されている[26-28]．日本人 2 型糖尿病患者にテネリグリプチンを 12 週間投与した治験では，本剤を 1 日 1 回 20 mg および 40 mg を投与した時の 12 週時における血中 DPP-4 阻害活性(当日は本剤投与前に測定)は各々 61.1 および 73.3%という結果が得られている[21]．しかしながら，血中 DPP-4 濃度が高いと DPP-4 阻害薬の血糖降下作用が減弱すると考えられており[27]，これらの DPP-4 活性を阻害するには高濃度の DPP-4 阻害薬を必要とする可能性がある．

テネリグリプチン 40 mg を 24 週間投与している間に，有害事象は 79.9%の患者で認められ，副作用は 7.4%の患者で認められたが，増量前と比較して発現頻度の明らかな増加は認められなかった．DPP-4 阻害薬と SU 薬との併用により低血糖のリスクが高まることが報告されている[24,29]．しかしながら，今回の検討では SU 薬の有無に関わらず，テネリグリプチン増量前後で低血糖の発現頻度に明らかな違いは認められなかった．

2 型糖尿病に対しては様々な治療薬が用いられている．患者の状態や薬剤の特性に基づき治療薬を選択することが重要である．高齢者や腎機能障害を有する患者のような場合には，治療の選択肢が少なくなることがある．テネリグリプチンは減量することなく腎機能低下患者(透析患者を含む)に処方することが可能である[30,31]．今回の結果は，糖尿病治療の選択肢が限られた患者も含め，糖尿病患者に対する治療の有用な情報となるかもしれない．今後，高用量のテネリグリプチンのベネフィットが最も得られる患者像の検討が必要である．

このポストホック解析にはいくつかのリミテーションがある．非盲検試験であり，サンプルサイズが小さい．加えて，心血管疾患リスクが高い患者や，重度の糖尿病性合併症を有する患者はこの治験から除外されている[24]．テネリグリプチンの増量を 28 週時から開始した患者であるため，血糖コントロールが早期に不良になった患者を対象としている可能性がある．この解析では，HbA1c の季節変動[32]およびテネリグリプチン増量後のテネリグリプチンや併用薬のアドヒアランスの変化を考慮していない．最後に，この解析ではインスリン，TZD，SGLT2 阻害薬などの糖尿病治療薬と併用した際のテネリグリプチンの評価は行っていない．

結論

このポストホック解析結果から，20 mg/日から 40 mg/日へのテネリグリプチンの増量は，日本人 2 型糖尿病患者に対して忍容性がある有効な選択肢となることが示唆された．
