# Supplementary material for: Efficacy and Safety of Teneligliptin 40 mg in Type 2 Diabetes: A Pooled Analysis of Two Phase III Clinical Studies
Source: Diabetes Ther. 2018 Feb 12;9(2):623–36. doi: 10.1007/s13300-018-0372-x (PMC6104275; doi:10.1007/s13300-018-0372-x)
Supplement: Supplementary file 2 — Supplementary material 2 (PDF 103 kb) [file 13300_2018_372_MOESM2_ESM.pdf]

Supplementary Figures and Tables

Figure S1 HbA1c levels over time

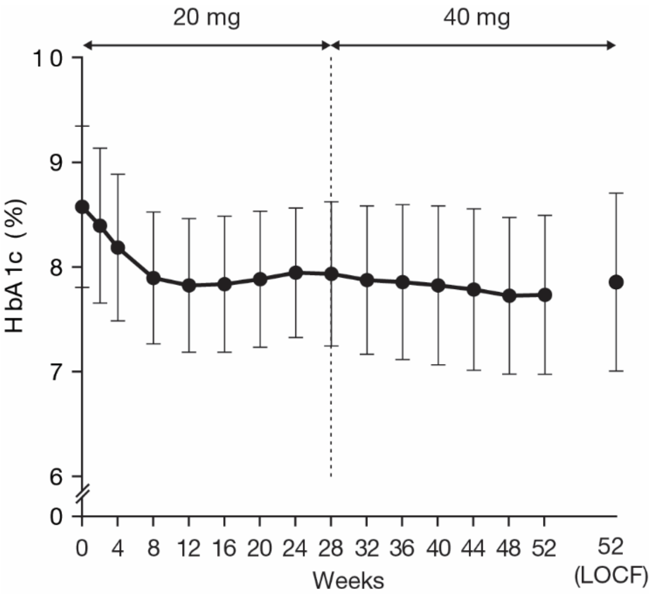

Data are mean ± SD; n=183–204

HbA1c glycated hemoglobin, *LOCF* last observation carried forward, *SD* standard deviation

Table S1 Fasting glucose and body weight at week 0, week 28, and week 52

	All (n = 204)	HbA1c ≤ – 0.1% (n = 108)	HbA1c > – 0.1% (n = 96)
Fasting glucose, mg/dL			
Week 0	171.0 (32.7)	172.1 (31.6)	169.7 (34.1)
Week 28	156.5 (27.4)	156.8 (26.1)	156.2 (29.0)
Week 52 (LOCF)	155.0 (35.7)	147.2 (31.7)	163.8 (37.9)
Δ 0–28 weeks	–14.5 (31.5)	–15.4 (30.0)	–13.5 (33.2)
Δ 0–52 weeks (LOCF)	–16.0 (34.7)	–25.0 (33.2)	–5.9 (33.7)
Δ 28–52 weeks (LOCF)	–1.5 (27.4)	–9.6 (25.6)	7.7 (26.6)
Body weight, kg			
Week 0	69.31 (14.87)	69.56 (15.28)	69.03 (14.47)
Week 28	70.08 (15.01)	70.21 (15.44)	69.94 (14.59)
Week 52 (LOCF)	69.65 (14.66)	69.28 (14.72)	70.06 (14.67)
Δ 0–28 weeks	0.77 (1.73)	0.65 (1.76)	0.91 (1.69)
Δ 0–52 weeks (LOCF)	0.34 (2.35)	–0.28 (2.56)	1.04 (1.86)
Δ 28–52 weeks (LOCF)	–0.43 (1.87)	–0.93 (2.20)[a]	0.13 (1.20)[a]

Data are mean (SD).

HbA1c glycated hemoglobin, *LOCF* last observation carried forward, *SD* standard deviation
[a] Based on results from logistic regression analysis, two sample *t*-test was performed between HbA1c ≤ – 0.1% subgroup and HbA1c > – 0.1% subgroup. $p < 0.0001$

Table S2 Adverse events occurring in ≥ 5% of patients (all patients, *n* = 204)

Number of patients with AE (%)	Weeks 0–52		Weeks 0–28 (Teneligliptin 20 mg)		Weeks 28–52 (Teneligliptin 40 mg)	
	n (%)	No. of events	n (%)	No. of events	n (%)	No. of events
Total	183 (89.7)	703	157 (77.0)	364	163 (79.9)	455
Bronchitis	17 (8.3)	19	11 (5.4)	11	9 (4.4)	10
Nasopharyngitis	64 (31.4)	89	44 (21.6)	51	35 (17.2)	42
Pharyngitis	14 (6.9)	14	5 (2.5)	5	11 (5.4)	11
Upper respiratory tract inflammation	22 (10.8)	29	16 (7.8)	19	10 (4.9)	13
Eczema	13 (6.4)	15	8 (3.9)	8	11 (5.4)	13
Blood creatine phosphokinase increased	16 (7.8)	18	10 (4.9)	10	9 (4.4)	9
Glucose urine present	31 (15.2)	41	19 (9.3)	22	21 (10.3)	26
Blood urine present	13 (6.4)	17	9 (4.4)	11	7 (3.4)	8
Protein urine present	23 (11.3)	29	13 (6.4)	13	18 (8.8)	21
Urine ketone body present	13 (6.4)	15	6 (2.9)	7	9 (4.4)	9

LIST OF INVESTIGATORS/INSTITUTIONS for the two studies for which data was pooled for the present analysis (3000-A8 and 3000-A14)

Atsushi Inoue (Japan Community Health Care Organization Hokkaido Hospital)

Yutaka Fujiwara (KKR Sapporo Medical Center)

Hirofumi Ohsaki (Medical Corporation Ohsaki Internal Medicine)

Takehiro Miyagishi (Miyagishi Clinic)

Toshiya Atsumi (Nissei Hospital)

Yuri Ono (Yuri Ono Clinic)

Shin Aoki (Aoki Clinic)

Yoshio Kurihara (Kurihara Diabetic Care Clinic)

Atsushi Hasegawa (Hasegawa Medicine Clinic)

Yoshihiko Kuroda (Takikawa Municipal Hospital)

Yuichi Nakamura (Nakamura Clinic)

Kazuo Yamagata (Sakajiri Clinic)

Satoshi Hayashi (Sunvillage Clinic)

Daisuke Koike (Hijirino Koike Clinic)

Daishiro Yamada (Jiyugaoka Yamada Clinic)

Shunya Sato (Foundation Shukokai Internal Medicine Sato Hospital)

Fuminobu Okuguchi (Okuguchi Clinic of Internal Medicine)

Kazuko Saito (Seiryo Medical Clinic)

Kazuhiko Sugiyama (Clinic Sugiyama)

Hiroshi Ohnuma (Sagae City Hospital)

Hiroshi Kouno (Jusendo General Hospital Yuasa Foundation)

Takeshi Osonoi (Nakakinen Clinic)

Eiko Onai (Hitachino Central Clinic)

Shinya Nakamoto (Nakamoto Medical Clinic)

Masakazu Mizutani (Kozawa Eye Hospital and Diabetes Center)

Hideo Takahashi (Minamiakatsuka Clinic)

Masayuki Noritake (Noritake Clinic)

Akira Ohishi (Ohishi Naika Clinic)

Koichi Taya (Taya Clinic)

Hiroshi Ohashi (Oyama East Clinic)

Tadashi Mugihara (Nogi Hospital)

Kazuo Aihara (Aihara Internal Medicine Clinic)

Yuko Miyazono (Miyazono Medical Clinic)

Masayoshi Sone (Sone Clinic)

Masaharu Morohoshi (Clinic for life-style related disease, Sanraku Hospital)

Fumihiko Hojo (Kobari General Clinic)

Osamu Funae (Tokui Medical Clinic)

Megumi Noguchi (Idaimae-naika clinic)

Hideo Takano (Takano Clinic)

Naoki Nomura (Tomei Atsugi Clinic)

Kiyokazu Matoba (Matoba Diabetes Clinic)

Madoka Taguchi (Toshiba General Hospital)

Genichi Watanabe (Watanabe Clinic of Internal Medicine)

Nobuo Takahashi (Takahashi Family Clinic)

Toshihiro Kojima (Kojima Internal Medicine Clinic)

Toshimitsu Sakai (Sakai medical clinic)

Masahiko Kushima (Kushima Internal Medicine Clinic)

Tetsuya Enomoto (Enomoto Internal Medicine Clinic)

Akira Yamauchi (Medical Care Law person corporation Rikeikai Suruga Clinic)

Kotaro Kawai (Shimada Municipal Hospital)

Eiji Oyake (Uzumasa Medical Clinic)

Hajime Fujita (Fujita Internal Medicine Clinic)

Kiyoshi Izumino (Fujikoshi Hospital)

Yukihiro Nagai (Nagai Internal Medicine Clinic)

Yuya Ueyama (Medical Corporation Nijunkai Ueyama Clinic)

Masaya Kino (Hokusetsu General Hospital)

Kiyoshi Takekawa (Takekawa Clinic)

Toshihiko Shiraiwa (Shiraiwa Medical Clinic)

Manabu Oya (Shinsuma General Hospital)

Akihiko Nakamura (Osafune Clinic)

Kenichi Kato (Yamana Medical Clinic)

Toshiki Fukui (Center for preventive medical treatment NTT west Takamatsu Hospital)

Akira Okada (Okada Clinic)

Hisao Ito (Ito Clinic)

Katsumi Noda (Clinic Tenjinkita)

Daisuke Goto (Kyushu Central Hospital of the Mutual Aid Association of Public School Teachers)

Makoto Kunisaki (Kunisaki Makoto Clinic, Medical Corporation Shinaikai)

Seiichi Tanaka (Kyushu Rosai Hospital)

Hidekatsu Sugimoto (Sugimoto Clinic)

Hideki Hayashi (Tenjinkai Koga Hospital 21)

Eiichi Higuchi (Nagata Hospital)

Kiyohiro Izumino (Shunkaikai Inoue Hospital)

Shinichiro Okuno (Okuno Clinic)

Kenichi Yamakawa (Yamakawa Clinic)

Miwa Izaki (Meiwakai Izaki Clinic)

Nobuyuki Abe (Abe Diabetes Clinic)

Takamoto Kodera (Saiki Central Hospital)

Masaru Doi (Doi Internal Medicine Clinic)

Mikio Todaka (Todaka Medical Clinic)

Ryohei Yoshimura (Yoshimura Clinic)

Takayuki Higashi (Higashi Diabetes and Cardiovascular Clinic)

Kouichi Sakai (Sakura Ganka Naika Clinic)

Shuji Nakamura (Iryouhoujinshadan Kouwakai Heiwadai Hospital)

Naoko Imamura (Jiaikai Clinic)

Noriko Nakamura (Primula Clinic)

Yoshihide Fukumoto (Fukumoto Clinic)
